# Challenges in the Diagnosis of Biliary Stricture and Cholangiocarcinoma and Perspectives on the Future Applications of Advanced Technologies

**DOI:** 10.3390/cancers17142301

**Published:** 2025-07-10

**Authors:** Kevin Gaston, Abdelkhalick Mohammad, Suresh Vasan Venkatachalapathy, Ioan Notingher, George S. D. Gordon, Arvind Arora, Frankie J. Rawson, Jane I. Grove, Abhik Mukherjee, Dhanny Gomez, Padma-Sheela Jayaraman, Guruprasad P. Aithal

**Affiliations:** 1 Biodiscovery Institute, School of Medicine, University of Nottingham, Nottingham NG7 2RD, UK; msaaa18@exmail.nottingham.ac.uk (A.A.); mszsj1@exmail.nottingham.ac.uk (P.-S.J.); 2 Mechanical, Material and Manufacturing Engineering, Faculty of Engineering, University of Nottingham, Nottingham NG7 2RD, UK; ezzamm@exmail.nottingham.ac.uk; 3 NIHR Nottingham Biomedical Research Centre, Nottingham University Hospitals NHS Trust, University of Nottingham, Nottingham NG7 2RD, UK; s.venkatachalapathy@nhs.net (S.V.V.); plzjig@exmail.nottingham.ac.uk (J.I.G.); mszam2@exmail.nottingham.ac.uk (A.M.); dhanny.gomez1@nhs.net (D.G.); mszag3@exmail.nottingham.ac.uk (G.P.A.); 4 School of Physics and Astronomy, University of Nottingham, Nottingham NG7 2RD, UK; ppzin@exmail.nottingham.ac.uk; 5 Optics and Photonics Research Group, Faculty of Engineering, University of Nottingham, Nottingham NG7 2RD, UK; ezzgsg@exmail.nottingham.ac.uk; 6 Laboratory of Bioelectronics, Regenerative Medicine & Cellular Therapies, School of Pharmacy, University of Nottingham, Nottingham NG7 2RD, UK; pazfr2@exmail.nottingham.ac.uk; 7 Nottingham Digestive Diseases Centre, Translational Medical Sciences, School of Medicine, University of Nottingham, Nottingham NG7 2RD, UK

**Keywords:** bile duct, cholangiocarcinoma, cholestasis, robotics, liver cancer

## Abstract

In the management of cholangiocarcinoma, effective biliary drainage and accurate diagnosis are vital to allow further treatment. Confirmation of tissue diagnosis and molecular characterization is also required to guide future treatment options including surgery and chemotherapy as well as the possible use of personalized treatments that target specific mutations present within individual tumours. Initial CT or MRI scans may be followed by endoscopic ultrasound (EUS) or endoscopic retrograde cholangiopancreatography (ERCP) to obtain tissue samples. However, these methods often fall short due to difficulty in accessing entire bile duct strictures. SpyGlass cholangioscopy can improve diagnosis, yet may fail to provide sufficient tissue for molecular characterization. Here we present a perspective on the development of snake-like agile robots with integrated optical imaging and Raman spectroscopy. These robots could improve the mapping of the biliary tree and the precision of biopsy collection and allow tissue analysis in situ, as well as facilitating stenting to restore the flow of bile. A multidisciplinary approach that brings together clinicians, pathologists, and engineers is required to develop these new robotic technologies and improve patient outcomes.

## Simple Summary

Cholangiocarcinoma, or bile duct cancer, can restrict the flow of bile from the liver to the gall bladder and intestine and cause jaundice. The restoration of biliary drainage using stents is required to enable further treatment, as is the collection of tumour samples that guide treatment options including surgery, chemotherapy, and targeted therapies. Computed Tomography (CT) X-ray imaging and magnetic resonance imaging (MRI) are initially used to image the biliary tract, and endoscopy can be used to obtain tissue samples for further characterization. However, these methods cannot fully map the biliary tract or adequately sample different parts of the duct. Here we describe how flexible robots could aid in navigating, mapping, and decompressing the blocked bile duct. Advanced imaging could also be incorporated into these robots to improve cancer detection and treatment. Collaboration between clinicians and engineers is required to develop these robotic tools and improve patient outcomes.

## 1. Introduction

Biliary strictures (abnormal narrowings in the biliary tract) present with cholestatic jaundice or cholangitis with manifestations such as itching, fever, and systemic inflammatory responses. Biliary stricture can occur throughout the biliary tree involving the intrahepatic, hilar, and extrahepatic biliary tract. The etiology of biliary strictures includes benign conditions, such as inflammation due to primary sclerosing cholangitis, scarring from bile duct stones, and ischemia, as well as malignant conditions such as cholangiocarcinoma (bile duct cancer), pancreatic cancer, and duodenal cancer. The diagnostic journey usually begins with cross-sectional imaging studies including CT and MRI scans which identify the extent of the narrowing along the axis of the biliary tree and the involvement of adjacent tissue or structures around the bile duct. Cholangiocarcinoma is classified as intrahepatic cholangiocarcinoma, which occurs within the liver, and hilar and extrahepatic cholangiocarcinoma which occur outside the liver where the left and right hepatic ducts merge and in the common bile duct, respectively [1,2]. However, despite recent advances in imaging methods, distinguishing benign from malignant strictures and accurately diagnosing cholangiocarcinoma still presents significant challenges [3,4,5]. Moreover, there is molecular heterogeneity within each cancer type that can only be assessed from tissue samples [6,7,8]. This review examines the complexities involved in performing endoscopic interventions for biliary strictures and acquiring tissue samples, underscoring how advancements in technology and methodology are vital to improving patient outcomes.

## 2. Diagnostic Techniques

Differential diagnosis of biliary strictures continues to be a challenge [4,5]. Abdominal ultrasound is the most accessible test and can demonstrate biliary obstruction based on the dilation of the biliary tree proximal to the obstruction, but this has low sensitivity in visualizing the cause of the blockage [9]. Almost all patients undergo CT or MRI scans to define the stricture; with MRI scans being superior to CT scans in defining the bile duct stricture. The sensitivity and specificity of defining a biliary stricture for Magnetic Resonance Cholangiopancreatography (MRCP) versus multi-detector CT are 88% versus 75% and 95% versus 60%, respectively [4,5,10].

Considering important differences in the management of benign strictures (which include primary sclerosing cholangitis and IgG4 sclerosing cholangitis) compared to cholangiocarcinoma, accurate diagnosis is sought using complementary modalities [11]. Once imaging is completed, the patient’s case is discussed at a multidisciplinary team meeting, where the best approach to obtaining tissue samples is determined. This might involve procedures such as Percutaneous Transhepatic Cholangiography (PTC), Endoscopic Ultrasound (EUS), or Endoscopic Retrograde Cholangiopancreatography (ERCP), each designed to obtain necessary tissue samples while also providing adequate biliary drainage [5,12]. Once invasive procedures such as ERCP or PTC are performed adequate biliary drainage is essential to reduce the risk of infection (cholangitis). Achieving effective biliary drainage is also desirable to permit further treatment [13,14]. Ideally, reduction in bilirubin levels to below 50 μmol/L is required to allow for a timely and meaningful intervention [15].

The standard first choice for tissue sampling is brush cytology, which often yields inconclusive results. The sensitivity of brush cytology for diagnosing biliary strictures is between 43 and 58% [11]. In cases where the initial sampling has been non-diagnostic, SpyGlass cholangioscopy can be performed for imaging and to capture biopsies using SpyBite biopsy forceps. This significantly increases sensitivity to 76.5–78% [16]. However, SpyGlass cholangioscopy has limited flexibility (with 2 degrees of movement), requires general anesthesia, and has limited accessibility in tertiary centres, hence there is a substantial selection bias in the reported sensitivity of this method [17].

## 3. Surgical Treatment Options

Surgical resection is the only potentially curative treatment for patients with cholangiocarcinoma. The American Joint Committee on Cancer (AJCC) staging system has classified cholangiocarcinoma to intrahepatic, perihilar and distal cholangiocarcinoma [1] due to the different tumour characteristics exhibited by each sub-type. These sub-types require different surgical approaches due to their anatomical location. Patients are initially staged with CT Chest, Abdomen and Pelvis. Current guidelines also recommend the use of CT-PET [2,3]. Following counselling and consenting for surgery, patients are enrolled in a pre-habilitation program [18] to improve their fitness and nutritional status before surgery.

Intrahepatic cholangiocarcinoma (ICCA): Following staging, unfortunately, the majority (70–80%) of ICCAs are diagnosed at an advanced, inoperable stage, and their management relies on best supportive care and chemotherapy depending on the patient’s fitness. Those deemed operable, will require liver resection. Although variation in practice exists [19], current guidelines also recommend portal lymphadenectomy [2,3]. The morbidity and mortality rates from liver resection with portal lymphadenectomy have improved over the years [19]. Nevertheless, patient selection is important to improve both short- and long-term outcomes.

Hilar cholangiocarcinoma: Similarly to ICCA, the majority of patients with hilar/perihilar CCA (sometimes known as Klatskin tumour) present at an inoperable stage. Pre-operative optimization involves improving biliary drainage and increasing the volume of the future liver remnant prior to surgery [20]. Most patients present with jaundice, necessitating either endoscopic or percutaneous drainage. Adequate nutritional support should also be provided in preparation for surgery. After biliary drainage, it is essential to determine the planned surgical approach, as this will inform the required volume of the liver remnant. If the estimated remnant volume is insufficient, liver augmentation procedures should be considered. Portal vein embolisation, with or without hepatic vein embolisation, may be employed to stimulate hypertrophy of the remnant liver [20].

Most patients will require major liver resection with portal lymphadenectomy, bile duct excision and reconstruction. This is a major liver operation that is associated with high morbidity and mortality [21], even in high volume centres [22]. Despite improvements in staging and treatment options, the overall survival of patients with hilar CCA undergoing surgery remains poor, with 5-year survival rates of less than 20% being reported [23]. This is partly related to the fact that most patients have high recurrence rates and this occurs within 12 months of resection [23,24].

Extrahepatic cholangiocarcinoma: Most patients with extrahepatic (or distal) CCA present with obstructive jaundice. These patients require urgent biliary drainage with an ERCP and stent insertion. In patients who are operable, they will require a Whipple’s procedure. Unfortunately, this procedure is associated with high morbidity [25]. In addition, recurrence rates remain high [25].

For patients who are stable enough for surgery, the surgical approach hinges on the tumour’s location:

• Extrahepatic tumours may be treated through the Whipple procedure which removes the pancreatic head, parts of the duodenum and bile duct, and the gallbladder.
• Intrahepatic tumours may be treated by surgical liver resection.
• Hilar tumours (at the Y-junction/bifurcation where the left and right hepatic ducts merge) require complex procedures that involve major hepatic resection.

However, surgical procedures come with their own risks, especially in cases of hilar cholangiocarcinoma, as about 80% of patients’ experience complications following major surgery, and the in-hospital mortality rate is around 15–20% [22]. Hence, it is paramount that we confirm an accurate diagnosis before surgical intervention.

More recently, liver transplantation has been proposed as a treatment option for unresectable hilar cholangiocarcinoma. With strict selection criteria and an aggressive neoadjuvant chemoradiation protocol, retrospective data from the Mayo Clinic group observed a 5-year survival rate of 82% for hilar cholangiocarcinoma patients without primary sclerosing cholangitis who underwent liver transplantation with a mortality rate of 8% [26]. With other centres reporting favourable outcomes [27,28,29,30], the U.S. Organ Procurement and Transplantation Network and United Network for Organ Sharing implemented a policy for Model for End-stage Liver Disease (MELD) exception scores for patients with hilar cholangiocarcinoma in the liver allocation system. The policy recognizes liver transplantation as a potential treatment option for patients with de novo locally advanced hilar cholangiocarcinoma. Similarly to resection cases, it is essential to accurately determine that patients have a histological diagnosis of cholangiocarcinoma prior to being considered for neoadjuvant chemoradiation and liver transplantation.

Emerging approaches to surgical and non-surgical treatment offer great promise. For example, histotripsy, which uses highly focused, high-frequency ultrasound waves to induce controlled acoustic cavitation to break down tumour tissue, is reported to be a safe and potentially effective treatment approach for liver tumours [31,32]. In addition, emerging imaging and sampling technology hold the promise of improving diagnostic accuracy. While initial costs may be higher, their efficiency in cutting down unnecessary procedures is likely to ease long-term patient care expenses. Speeding up diagnosis should lead to early treatment, preventing what are otherwise operable cancers from becoming inoperable [33].

## 4. Histopathology

Histopathological samples are the backbone of cholangiocarcinoma treatment and research. They are essential for diagnosis and precision medicine as well as for advancing translational therapeutics. However, cholangiocarcinoma research has not advanced as rapidly as that for other cancers due in part to the narrow and intricate anatomy of the biliary system; there are challenges related to location and heterogeneity within the biliary tree, and in acquiring samples from difficult-to-reach areas. Moreover, a balance must be struck between patient care and research demands, ensuring that our histopathological sampling is robust. For instance, cytology is often compromised by sample adequacy, making accurate diagnosis challenging, particularly in fibrotic/mucinous/inflamed tumours. Indeed, indefinite/suspicious only diagnosis often necessitates a cycle of repeat sampling to reach a definitive diagnosis. While mass-forming cholangiocarcinoma is easier to sample, the mixed intraductal and periductal forms present significant difficulties. Where an accurate assessment of depth of invasion is essential for prognostication (T staging or surgical/circumferential margin assessment), it is difficult to spare fresh tissue for freezing as the deepest point of tumour invasion is not often discernable macroscopically and hence demands blocking out the entire field, to prevent understaging.

Cholangiocarcinoma comes in various histological patterns, from poorly differentiated forms to mucinous and clear cell types, adding complexity to diagnosis and staging. Fibrogenic tumours may produce a low tumour cell yield on sampling and inflammation/stenting induced atypia may be difficult to distinguish from malignant atypia. Premalignant lesions, for example, conventional dysplasia, intraductal tubulopapillary/papillary lesions of the bile ducts (ITPN-Bs/IPNB) may co-exist with invasive areas and may complicate sampling. Optical and imaging techniques, along with molecular studies, may offer insights into the characteristics of normal, dysplastic, and malignant tissues. However, fresh frozen samples for these studies are not always obtainable as dictated by patient staging priorities and hence scrolls from paraffin embedded tissue may be a reasonable solution.

**Figure 1:**
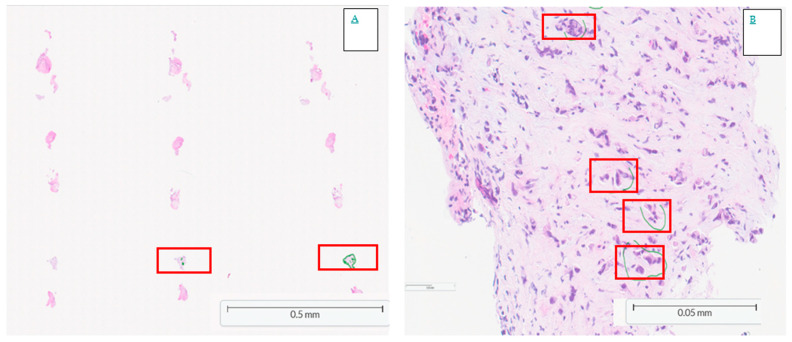
(**A**) Haematoxylin and eosin-stained histology of a SpyBite sample from bile duct stricture ×40 magnification. Only 1 fragment had cancer cells (red rectangle), magnified ×400 in (**B**). This illustrates that while low tumour content does not restrict diagnosis, further molecular studies and research sample availability is constrained. Green lines indicate pathologist highlighted areas.

Although the mutational profiles of intrahepatic and extrahepatic cholangiocarcinoma are similar recent studies have identified important molecular variations [6]. Intrahepatic cholangiocarcinomas are more frequently associated with IDH1 mutations [34] and FGFR fusions [7,8], which carry therapeutic implications as discussed further below. In a developing era of molecular therapeutics, even if a low percentage of malignant cells in a sample has sufficed for an unequivocal diagnosis of malignancy, careful macro-dissection may be required to achieve the limit of detection for further molecular testing. Such concerns may constrain tissue sparing for research to fulfil the requirements of the care pathway. An example is illustrated in Figure 1.

## 5. Oncology

We are moving from classification of tumours purely based on their anatomy to a more nuanced molecular classification that shapes treatment strategies with approved targeted treatments for patients based on specific molecular alterations (see Table 1). For example, tumours harbouring *NTRK* fusions now have targeted options such as Larotrectinib and Entrectinib, while tumours with *FGFR2* fusions can be treated with Pemigatinib and Futibatinib, and tumours carrying *IDH1* mutations can be treated with Ivosidenib [35,36,37]. Moreover, Pembrolizumab can be effective for MMR-deficient tumours [38].

If a patient has resectable disease they typically receive 6 months of adjuvant chemotherapy with Capecitabine based on data from the BILCAP study [50]. If they progress to metastatic disease or present with *de novo* locally advanced or metastatic disease, it becomes vital to perform molecular testing at the earliest opportunity. Detecting alterations early can allow us to offer targeted treatment plans, with around 40% of these patients possessing qualifying genetic alterations.

New combinations involving chemotherapy and immunotherapy have also borne fruit. For example, in the Topaz 1 study, after two years, 25% of patients who received the combination chemotherapy and immunotherapy were still alive compared to just 12% in the traditional chemotherapy group, and after 3-year follow-up, 14.6% of patients who received the combination chemotherapy and immunotherapy remained alive compared with only 6.9% of those who received chemotherapy [51]. This signifies a turning point in global treatment standards.

For patients with *FGFR* fusion positive CCA, Pemigatinib significantly improved survival rates (median overall survival (mOS) 17.5 m) in the FIGHT-202 study [39]. Similarly, for *IDH1* mutated intrahepatic CCA patients, Ivosidenib showed notable improvements in survival (mOS 10.6 m) in the ClarIDHy trial [35]. In contrast, traditional standard second line chemotherapy with FOLFOX showed mOS of 6.2 months in the ABC-06 study [52]. MMR-deficiency is rare, yet these patients can respond well to immunotherapies—illustrated by a 34% response rate and a median survival of 23.5 months reported in the KEYNOTE-158 study [53]. Advances in treatment also cater to tumours with other specific mutations, such as *BRAF* V600E, which have shown benefits from targeted therapy with Dabrafenib plus Trametinib in patients with *BRAF* V600E-mutated biliary tract cancer in a Phase 2 ROAR study [44].

Given that identifying genetic alterations is fundamental for enhancing patient outcomes we must ensure we have adequate tissue samples to pinpoint these mutations. Moreover, the earlier we can detect these alterations—ideally before first-line therapy—the better it will be for patients. The use of circulating tumour DNA (CtDNA) to identify actionable mutations is under intensive investigation and has been comprehensively reviewed recently [54]. However, looking to the future, there is the potential to adapt phenotypic and mapping techniques that could predict mutations without the need for tissue samples and this could fundamentally reshape our approach to diagnosis and treatment.

## 6. A Perspective on Next Generation Technologies

To overcome the challenges with diagnosis, sampling of biliary strictures, and molecular characterization of cholangiocarcinoma, we need to develop new technologies. In a research programme co-created by an interdisciplinary team of clinicians and scientists from diverse disciplines we are developing four different technologies: (i) An agile and slender robot that can pass through the channel of the current endoscope and is designed to navigate the narrowed segment of the bile duct. (ii) Fringe projections from this robot with devices to provide 3 dimensional axial spatial forward imaging of the stricture [55,56]. (iii) Raman spectroscopy to map the stricture and create maps based on molecular signatures and encompassing tumour heterogeneity [57]. (iv) Improved stent placement and coated stents for enhanced biliary drainage and the ability to deliver local therapy.

A snake-like agile robot for endoscopic navigation: Traditional endoscopes are limited to two degrees of freedom (DoF)—up/down and left/right— and this hinders smooth navigation of the biliary tract. Furthermore, they lack endoscope shape sensing, which is crucial for gauging shape within the bile ducts. Additionally, their manual handling via knobs proves impractical when increased flexibility is required. Similar challenges arise in the inspection and repair of capital-intensive infrastructures, such as aircraft, power plants, and telecommunication networks. These operations often involve navigating narrow, complex environments with limited direct access. For example, components within a gas turbine are accessible only through narrow channels like borescope ports, with reach extending several meters [58]. Conventional borescopes (optical instruments with two DoF consisting of a rigid or flexible tube with a lens or camera connected to an eyepiece or display) are currently the standard solution for in situ inspection in these contexts. However, they possess significant limitations: firstly, their limited tip bending control is insufficient for effective defect intervention; secondly, difficulty in positioning the borescope camera relative to the inspection target results in low defect detection rates [59]; and finally, manual operation requires a skilled operator, and even then, the process is slow and hindered by the inability to control the shape of the tool. To address these issues, a team at the Rolls-Royce University Technology Center at the University of Nottingham developed the continuum (snake-like) robot (COBRA) system [59], a 5-meter-long snake-like robot with a 6-DoF active section, capable of navigating through engines for inspection and repair. Compared to borescopes, continuum robots offer a significant advantage through their shape control, enabling intervention and substantially improving navigation. COBRA features a comprehensive actuation unit with motorized control and a user-friendly interface, incorporating a “twist-and-feed” mechanism that mimics human hand movements for precise guidance. Actuated by steel cables controlled via motors, it provides superior flexibility relative to standard endoscopes with six degrees of freedom and enhanced maneuverability. The robot also integrates tools such as cameras and laser cutters.

Inspired by COBRA, we propose a 3.5 mm snake-like robot with 6 DoF for improved navigation within the bile duct. This proposed CholangioBotics snake robot comprises a passive section that naturally follows the leading section [59], and an active segment that enables controlled movement with 6 DoF. We initially aim for a 3.5 mm diameter, with future designs targeting 2 mm for enhanced accessibility. The robot will feature a user-friendly joystick and screen interface for intuitive control and it may extend over a meter in length, allowing for deep navigation within the bile ducts. Furthermore, it will integrate advanced sensors and imaging tools for detailed tissue analysis. This robotic system represents a significant advancement over conventional endoscopic methods, enhancing flexibility, precision, and automation in complex medical procedures.

Improved vision for 3D spatial mapping and cancer triaging: Giving the agile robot “Eyes”: Effective navigation and biopsy in the bile duct require high-quality imaging within extremely confined spaces, demanding devices with ultra-thin form factors and high-resolution, high-contrast capabilities. These constraints, common to other domains such as pancreatic cyst imaging [60,61], are driving the development of advanced optical technologies. Optical fibres, with diameters as small as 0.1 mm—significantly smaller than the smallest commercial cameras (~1 mm)—enable the creation of hair-thin imaging devices [62,63]. These are already in use in neuroscience for deep brain imaging in animal models (for example Modendo, Deepen, Inscopix, and Transcend Vivoscope). In endoscopy, commercially available systems such as the SpyGlass DSII (Boston Scientific Corp, Natick, MA, USA) (3.3 mm, white light) and CellVizio (0.9 mm, confocal laser endomicroscopy) (Mauna Kea Technologies, Paris, France) demonstrate the clinical value of high-resolution imaging but remain limited in modality and miniaturization.

For next-generation diagnostics, advanced fibre-based probes must support multiple imaging modalities to exploit different biological contrast mechanisms. Modern microscopy relies on a variety of contrast techniques—fluorescence, phase contrast, dark/bright field, polarization, super-resolution, and multiphoton imaging—but these are challenging to miniaturize. Nonetheless, recent breakthroughs have demonstrated fibre-based implementations of fluorescence [64], super-resolution [62], and phase, polarization, and OCT imaging [65].

Of relevance to cholangiocarcinoma is the development of 3D structured illumination imaging. We have developed a 3 mm diameter prototype device that projects engineered light patterns onto tissue to extract absorption (light/dark contrast), scattering (surface roughness), and 3D texture information—each of which has been independently validated for cancer detection in tissues such as pancreas, colon, esophagus, and skin [66,67,68,69]. No existing system combines all three features in such a compact endoscopic format while offering a wide field-of-view necessary for guiding more targeted biopsies [68]. This multimodal, wide-field approach offers enhanced contrast by exploiting cancer-specific absorption and scattering properties, supporting early detection and triaging of biliary strictures. The integration of structured illumination allows for shape mapping to provide morphological context and potential cross-correlation with other modalities [55,56]. Preliminary testing on synthetic tissue models—designed to mimic varying optical properties of biological tissues—has shown substantial contrast improvement over standard imaging. Structured illumination at the robotic tip will enable real-time 3D mapping for navigation and tumour localization, laying the groundwork for a minimally invasive, highly effective diagnostic tool for cholangiocarcinoma.

Integrating Raman spectroscopy: giving the agile robot “ears”: Raman spectroscopy is based on inelastic optical scattering, a phenomenon in which laser photons incident on a sample are inelastically scattered after interacting with vibrating molecules within the sample. Thus, incorporating Raman spectroscopy on the robot probe will allow it to “hear” by essentially tuning in to the vibrations of molecules present in tissues. Every molecule has a distinct vibrational fingerprint and Raman spectroscopy can measure the concentrations of biomolecules such as lipids, proteins, carbohydrates, and nucleic acids [70]. The high accuracy for medical diagnosis comes from the ability to detect small changes in the relative concentrations of these biomolecules within a cell or tissue without labelling or predetermined biomarkers or any other prior information [70]. However, the molecular complexity of tissue leads to complex Raman spectra, necessitating sifting through a large volume of data to glean meaningful information. Raman spectroscopy alone does not reveal what is present unless we compare findings against a reference database. To accurately pinpoint molecules within tissues to provide useful diagnosis, it is therefore imperative to build a classification model. Once the system is trained (typically using machine learning), it becomes capable of real-time analysis and diagnosis of tissue samples.

One noteworthy application we are developing involves assessing surgical margins to help surgeons determine if any cancerous cells linger post-excision and remove any remaining tissue if needed [71]. In the training phase we collect tissue samples with known histology, capturing the respective Raman spectra from varying tissue types and training a classifier to distinguish between healthy and cancerous varieties. In real-time testing the system analyzes newly collected tissue samples without requiring pre-labelled histology, comparing the Raman data against the trained model to create colour-coded maps highlighting potential cancerous areas. Thus far, we have tested this method on significant surgical resections, including skin cancer [72,73], colorectal liver metastases [74], breast cancer excisions [70], and sentinel lymph node biopsies [75]. In recent studies using the first prototype in the clinic surgeons have utilized this system to examine tissue with surprising efficacy within just 40 min of excision—post-operation, and with no labelling or sectioning. Results from our tests indicated 96% sensitivity when samples were correctly positioned, and 73% specificity [75,76]. Comparable outcomes to frozen section analysis as performed during Mohs surgeries (86% sensitivity, higher specificity). The technique’s success extended to breast tissues and lymph nodes, demonstrating its value in prompt cancer detection.

For the effective integration of Raman spectroscopy within the snake robot, we require an ultra-small fibre probe. The narrowest Raman probes working in the fingerprint spectral region are 1–2 mm diameter, and there is a challenge in reducing the diameter to match the space in the robot. However, employing such a probe during initial tests will enable us to refine this technology ahead of its miniaturization.

So far, our expertise has centred on Raman systems used in microscopy for surgical analysis. The next logical step would be to pinpoint the necessary molecular sensitivity and specificity and to tailor our techniques for use in miniaturized and real-time contexts. An important aspect to consider is the sequence of imaging steps. Raman spectroscopy is a point-and-shoot technique. Thus, the approach could involve wide-field imaging to identify regions of interest followed by the application of Raman spectroscopy to pursue specific areas in detail. This two-step approach may significantly enhance both the accuracy and efficiency of the detection method.

## 7. Improving Stenting

Several biliary tract disorders require stenting including inflammatory strictures, ductal stones and bile duct and gall bladder cancer. Currently there are broadly two types of stent in clinical use plastic stents and self-expanding metal stents (SEMs). Plastic stents are often used because of their easy insertion and removal and lower cost compared to a SEM. SEMs are often made of an alloy of Nickel and titanium (Nitinol) that retains shape memory. SEMs increase the luminal diameter to a greater extent than a plastic stent and they are less likely to move within the duct once positioned [77]. Biodegradable stents are being developed and will be useful in benign conditions [78]. However, stents are prone to migration after placement, biofilm formation, and tumour in-growth all of which decrease patency.

Tumour in-growth: SEMs are prone to tumour cell in-growth as well as tumour overgrowth around the ends of the stent. To overcome this the SEMs have been either partly or fully covered with Silicone, polyurethane, or expanded polytetrafluoroethylene coverings [79,80]. Tumour in-growth is also being tackled through the development of covered drug eluting biliary stents. Paclitaxel (a microtuble inhibitor) elution from a polyurethane coated metal stent was proven to have efficacy against tumour in-growth in animal models and was also investigated in a pilot study in 21 patients with unresectable malignant biliary obstruction and shown to be safe and to have some efficacy against the in growth of the tumour [81]. Gemcitabine, a cytidine analogue and potent chemotherapeutic for biliary malignancy has also been investigated for local elution from a covered metal stent. In a pig model the elution of Gemcitabine was safe but treatment was accompanied by bile duct hyperplasia which needs further investigation [82]. Other kinds of biliary DESs, such as, sorafenib-eluting, and vorinostat-eluting stents, have also been reported [83,84]. Gemcitabine and/or Cisplatin coated stents also showed a sustained local drug release and potent antitumor activity in animal common bile duct models and importantly the stent placement resulted in little bile duct hyperplasia [85].

Biofilm formation: Both plastic and metal stents can become occluded with bacterial biofilm. Stent occlusion starts with the deposition of biliary sludge consisting of cholesterol crystals, calcium bilirubinate and palmitate, glycoproteins, bacteria and/or fungi. The adhesion of micro-organisms to the proteins coating the stent is thought to be a major contributor to sludge formation. Silver nanoparticles have been coated onto SEMS by attaching them to chitosan and then layering onto polyester membrane to produce a fully coated SEM stent and in preclinical studies was effective as a means to prevent the growth of bacteria and improve stent patency [86,87].

Stent movement: The positioning of a stent is generally assessed by Computed Tomography (CT). Biliary stents made of metals (such as Nitinol or stainless-steel alloys) can be observed by CT but the contours of the stent are not clearly visible. Coatings that increase their visibility are being developed. For example, Nitinol stents have been covered with a silicone membrane sandwiching the metal Tantalum which increases the visualization of the stent to X-rays [88]. Coatings that decrease stent movement are also being developed such as stents with membranes with small holes to allow bile duct flow improves implantation into the bile duct wall [89]. Flexible robotic technology could improve mapping of the stricture and stent placement and thereby help to reduce stent movement.

## 8. Ethically Obtaining Samples for Molecular Mapping and Research

The development of preclinical models such as ex vivo cell cultures and testing of emerging technologies and devices requires a substantial supply of fresh characterized patient tissue, representative of diverse cancer phenotypes, obtained ethically from clinical procedures locally. This can only be achieved through cross-disciplinary collaboration and careful sample management prioritizing samples for clinical diagnostics and patient care. Standard endoscopic or radiolological procedures can safely provide additional research samples but these should be held as available for diagnostics to avoid resampling when diagnostic samples are inadequate [90,91]. However, although tissue from investigations is readily available, this yields very small quantities. Alternatively, tissues from surgeries can provide larger quantities but suitable cases are uncommon and require prior identification by the clinical multidisciplinary team. However, these samples are only available after lengthy systematic macroscopic evaluation and sampling by the pathologist is completed. Non-destructive technologies such as Raman spectroscopy have the opportunity to assess diagnostic tissues close to ‘bedside’ before, and without delaying, the usual diagnostic processing pathway, thus providing supplemental information supporting diagnosis [92].

## 9. Discussion: Engineering Life for Advancing Care

Navigating the complex anatomy and challenges posed by biliary stricture is an important step in advancing care for patients with cholangiocarcinoma. Emerging such as super-slender, agile robot, optical spatial imaging and molecular mapping promise to bring about necessary step change in the lives of patients. Collaborating closely between clinicians and researchers ensures that emerging solutions are not only innovative but also clinically relevant and actionable in practice. Improved diagnostic accuracy and integration of molecular characteristics into personalized treatment plans will improve life span and the quality of life for patients with biliary strictures.

## 10. Conclusions

The future of diagnosing and treating cholangiocarcinoma hinges on advancements in imaging, robotics, and molecular research. While current methods such as ERCP, PTC, and SpyGlass endoscopy have improved our ability to detect and manage bile duct strictures, limitations in diagnostic accuracy and accessibility remain significant hurdles. The integration of robotic endoscopy, structured illumination imaging, and Raman spectroscopy presents an opportunity to refine early detection and precision in biopsy collection. Moreover, molecular testing has revolutionized our understanding of tumour classifications, leading to targeted therapies that promise more effective treatment outcomes. As technology evolves, minimizing surgical risks and reducing unnecessary interventions will be of paramount importance. The development of next-generation diagnostic tools, including flexible robotic endoscopes and improved stenting techniques, could transform patient care, making timely and accurate diagnosis the standard rather than the exception. While challenges persist—ranging from sample collection difficulties to the need for widespread adoption of emerging technologies—the collaborative efforts of pathologists, oncologists, engineers, and researchers signal a promising shift towards more personalized and effective treatments to improve survival rates and quality of life.

## Figures and Tables

**Table 1:** Selected targeted drug treatments with applications in cholangiocarcinoma.

Mutated Gene(s)	Drugs	References
*FGFR2*	Pemigatinib Futibatinib Infigratinib Erdafitinib	[37,39,40,41]
*IDH1*	Ivosidenib	[35,36]
*NTRK family*	Larotrectinib Entrectinib Repotrectinib	[42,43]
*BRAF*	Dabrafenib Trametinib	[44]
*RET*	Selpercatinib Pralsetinib	[45,46]
*HER2*	Zanidatamab Trastuzumab deruxtecan	[47,48,49]

## Data Availability

Not applicable.

## References

[1] Blechacz B., Komuta M., Roskams T., Gores G.J. (2011). Clinical diagnosis and staging of cholangiocarcinoma. Nat. Rev. Gastroenterol. Hepatol..

[2] Valle J.W., Borbath I., Khan S.A., Huguet F., Gruenberger T., Arnold D., Committee E.G. (2016). Biliary cancer: ESMO Clinical Practice Guidelines for diagnosis, treatment and follow-up. Ann. Oncol..

[3] Rushbrook S.M., Kendall T.J., Zen Y., Albazaz R., Manoharan P., Pereira S.P., Sturgess R., Davidson B.R., Malik H.Z., Manas D. (2023). British Society of Gastroenterology guidelines for the diagnosis and management of cholangiocarcinoma. Gut.

[4] Saluja S.S., Sharma R., Pal S., Sahni P., Chattopadhyay T.K. (2007). Differentiation between benign and malignant hilar obstructions using laboratory and radiological investigations: A prospective study. HPB.

[5] Rosch T., Meining A., Fruhmorgen S., Zillinger C., Schusdziarra V., Hellerhoff K., Classen M., Helmberger H. (2002). A prospective comparison of the diagnostic accuracy of ERCP, MRCP, CT, and EUS in biliary strictures. Gastrointest. Endosc..

[6] Roos E., Soer E.C., Klompmaker S., Meijer L.L., Besselink M.G., Giovannetti E., Heger M., Kazemier G., Klumpen H.J., Takkenberg R.B. (2019). Crossing borders: A systematic review with quantitative analysis of genetic mutations of carcinomas of the biliary tract. Crit. Rev. Oncol. Hematol..

[7] Arai Y., Totoki Y., Hosoda F., Shirota T., Hama N., Nakamura H., Ojima H., Furuta K., Shimada K., Okusaka T. (2014). Fibroblast growth factor receptor 2 tyrosine kinase fusions define a unique molecular subtype of cholangiocarcinoma. Hepatology.

[8] Graham R.P., Barr Fritcher E.G., Pestova E., Schulz J., Sitailo L.A., Vasmatzis G., Murphy S.J., McWilliams R.R., Hart S.N., Halling K.C. (2014). Fibroblast growth factor receptor 2 translocations in intrahepatic cholangiocarcinoma. Hum. Pathol..

[9] Vardar B.U., Dupuis C.S., Goldstein A.J., Vardar Z., Kim Y.H. (2022). Ultrasonographic evaluation of patients with abnormal liver function tests in the emergency department. Ultrasonography.

[10] Romagnuolo J., Bardou M., Rahme E., Joseph L., Reinhold C., Barkun A.N. (2003). Magnetic resonance cholangiopancreatography: A meta-analysis of test performance in suspected biliary disease. Ann. Intern. Med..

[11] Hori Y., Chari S.T., Tsuji Y., Takahashi N., Inoue D., Hart P.A., Uehara T., Horibe M., Yamamoto S., Satou A. (2021). Diagnosing Biliary Strictures: Distinguishing IgG4-Related Sclerosing Cholangitis From Cholangiocarcinoma and Primary Sclerosing Cholangitis. Mayo Clin. Proc. Innov. Qual. Outcomes.

[12] Raza D., Singh S., Crino S.F., Boskoski I., Spada C., Fuccio L., Samanta J., Dhar J., Spadaccini M., Gkolfakis P. (2025). Diagnostic Approach to Biliary Strictures. Diagnostics.

[13] Dumonceau J.M., Tringali A., Papanikolaou I.S., Blero D., Mangiavillano B., Schmidt A., Vanbiervliet G., Costamagna G., Deviere J., Garcia-Cano J. (2018). Endoscopic biliary stenting: Indications, choice of stents, and results: European Society of Gastrointestinal Endoscopy (ESGE) Clinical Guideline-Updated October 2017. Endoscopy.

[14] Venkatachalapathy S.V., James M.W., Huggett M.T., Paranandi B., Pereira S.P., Johnson G., Aravinthan A.D., Aithal G.P. (2021). Utility of palliative EUS-guided biliary drainage using lumen-apposing metal stents: A prospective multicenter feasibility study (with video). Gastrointest. Endosc..

[15] Field K.M., Michael M. (2008). Part II: Liver function in oncology: Towards safer chemotherapy use. Lancet Oncol..

[16] Stassen P.M.C., Goodchild G., de Jonge P.J.F., Erler N.S., Anderloni A., Cennamo V., Church N.I., Fernandez-Urien Sainz I., Huggett M.T., James M.W. (2021). Diagnostic accuracy and interobserver agreement of digital single-operator cholangioscopy for indeterminate biliary strictures. Gastrointest. Endosc..

[17] Subhash A., Abadir A., Iskander J.M., Tabibian J.H. (2021). Applications, Limitations, and Expansion of Cholangioscopy in Clinical Practice. Gastroenterol. Hepatol..

[18] Skorepa P., Ford K.L., Alsuwaylihi A., O’Connor D., Prado C.M., Gomez D., Lobo D.N. (2024). The impact of prehabilitation on outcomes in frail and high-risk patients undergoing major abdominal surgery: A systematic review and meta-analysis. Clin. Nutr..

[19] McClements J., Valle J.W., Blackburn L., Brooks A., Prachalias A., Dasari B.V.M., Jones C., Harrison E., Malik H., Prasad K.R. (2023). Variation in treatment of intrahepatic cholangiocarcinoma: A nationwide multicentre study. Br. J. Surg..

[20] Hewitt D.B., Brown Z.J., Pawlik T.M. (2022). Current Perspectives on the Surgical Management of Perihilar Cholangiocarcinoma. Cancers.

[21] Franken L.C., Schreuder A.M., Roos E., van Dieren S., Busch O.R., Besselink M.G., van Gulik T.M. (2019). Morbidity and mortality after major liver resection in patients with perihilar cholangiocarcinoma: A systematic review and meta-analysis. Surgery.

[22] Mueller M., Breuer E., Mizuno T., Bartsch F., Ratti F., Benzing C., Ammar-Khodja N., Sugiura T., Takayashiki T., Hessheimer A. (2021). Perihilar Cholangiocarcinoma-Novel Benchmark Values for Surgical and Oncological Outcomes From 24 Expert Centers. Ann. Surg..

[23] Zhang X.F., Beal E.W., Chakedis J., Chen Q., Lv Y., Ethun C.G., Salem A., Weber S.M., Tran T., Poultsides G. (2018). Defining Early Recurrence of Hilar Cholangiocarcinoma After Curative-intent Surgery: A Multi-institutional Study from the US Extrahepatic Biliary Malignancy Consortium. World J. Surg..

[24] Groot Koerkamp B., Wiggers J.K., Allen P.J., Besselink M.G., Blumgart L.H., Busch O.R., Coelen R.J., D’Angelica M.I., DeMatteo R.P., Gouma D.J. (2015). Recurrence Rate and Pattern of Perihilar Cholangiocarcinoma after Curative Intent Resection. J. Am. Coll. Surg..

[25] Labib P.L., Russell T.B., Denson J.L., Puckett M.A., Ausania F., Pando E., Roberts K.J., Kausar A., Mavroeidis V.K., Bhogal R.H. (2024). Patterns, timing and predictors of recurrence following pancreaticoduodenectomy for distal cholangiocarcinoma: An international multicentre retrospective cohort study. Eur. J. Surg. Oncol..

[26] Tan E.K., Taner T., Heimbach J.K., Gores G.J., Rosen C.B. (2020). Liver Transplantation for Peri-hilar Cholangiocarcinoma. J. Gastrointest. Surg..

[27] De Vreede I., Steers J.L., Burch P.A., Rosen C.B., Gunderson L.L., Haddock M.G., Burgart L., Gores G.J. (2000). Prolonged disease-free survival after orthotopic liver transplantation plus adjuvant chemoirradiation for cholangiocarcinoma. Liver Transpl..

[28] Sudan D., DeRoover A., Chinnakotla S., Fox I., Shaw B., McCashland T., Sorrell M., Tempero M., Langnas A. (2002). Radiochemotherapy and transplantation allow long-term survival for nonresectable hilar cholangiocarcinoma. Am. J. Transplant..

[29] Ahmed O., Vachharajani N., Chang S.H., Park Y., Khan A.S., Chapman W.C., Doyle M.B.M. (2022). Single-center experience of liver transplantation for perihilar cholangiocarcinoma. HPB.

[30] Darwish Murad S., Kim W.R., Harnois D.M., Douglas D.D., Burton J., Kulik L.M., Botha J.F., Mezrich J.D., Chapman W.C., Schwartz J.J. (2012). Efficacy of neoadjuvant chemoradiation, followed by liver transplantation, for perihilar cholangiocarcinoma at 12 US centers. Gastroenterology.

[31] Mendiratta-Lala M., Wiggermann P., Pech M., Serres-Creixams X., White S.B., Davis C., Ahmed O., Parikh N.D., Planert M., Thormann M. (2024). The #HOPE4LIVER Single-Arm Pivotal Trial for Histotripsy of Primary and Metastatic Liver Tumors. Radiology.

[32] Wehrle C.J., Burns K., Ong E., Couillard A., Parikh N.D., Caoili E., Kim J., Aucejo F., Schlegel A., Knott E. (2025). The first international experience with histotripsy: A safety analysis of 230 cases. J. Gastrointest. Surg..

[33] de Jong D.M., de Jonge P.J.F., Stassen P.M.C., Karagyozov P., Vila J.J., Fernandez-Urien I., James M.W., Venkatachalapathy S.V., Oppong K.W., Anderloni A. (2025). The value of cholangioscopy-guided bite-on-bite (-on bite) biopsies in indeterminate biliary duct strictures. Endoscopy.

[34] Borger D.R., Tanabe K.K., Fan K.C., Lopez H.U., Fantin V.R., Straley K.S., Schenkein D.P., Hezel A.F., Ancukiewicz M., Liebman H.M. (2012). Frequent mutation of isocitrate dehydrogenase (IDH)1 and IDH2 in cholangiocarcinoma identified through broad-based tumor genotyping. Oncologist.

[35] Abou-Alfa G.K., Macarulla T., Javle M.M., Kelley R.K., Lubner S.J., Adeva J., Cleary J.M., Catenacci D.V., Borad M.J., Bridgewater J. (2020). Ivosidenib in IDH1-mutant, chemotherapy-refractory cholangiocarcinoma (ClarIDHy): A multicentre, randomised, double-blind, placebo-controlled, phase 3 study. Lancet Oncol..

[36] Lowery M.A., Burris H.A., Janku F., Shroff R.T., Cleary J.M., Azad N.S., Goyal L., Maher E.A., Gore L., Hollebecque A. (2019). Safety and activity of ivosidenib in patients with IDH1-mutant advanced cholangiocarcinoma: A phase 1 study. Lancet Gastroenterol. Hepatol..

[37] Goyal L., Meric-Bernstam F., Hollebecque A., Valle J.W., Morizane C., Karasic T.B., Abrams T.A., Furuse J., Kelley R.K., Cassier P.A. (2023). Futibatinib for FGFR2-Rearranged Intrahepatic Cholangiocarcinoma. N. Engl. J. Med..

[38] Maio M., Ascierto P.A., Manzyuk L., Motola-Kuba D., Penel N., Cassier P.A., Bariani G.M., De Jesus Acosta A., Doi T., Longo F. (2022). Pembrolizumab in microsatellite instability high or mismatch repair deficient cancers: Updated analysis from the phase II KEYNOTE-158 study. Ann. Oncol..

[39] Abou-Alfa G.K., Sahai V., Hollebecque A., Vaccaro G., Melisi D., Al-Rajabi R., Paulson A.S., Borad M.J., Gallinson D., Murphy A.G. (2020). Pemigatinib for previously treated, locally advanced or metastatic cholangiocarcinoma: A multicentre, open-label, phase 2 study. Lancet Oncol..

[40] Bekaii-Saab T.S., Valle J.W., Van Cutsem E., Rimassa L., Furuse J., Ioka T., Melisi D., Macarulla T., Bridgewater J., Wasan H. (2020). FIGHT-302: First-line pemigatinib vs gemcitabine plus cisplatin for advanced cholangiocarcinoma with FGFR2 rearrangements. Future Oncol..

[41] Javle M., Roychowdhury S., Kelley R.K., Sadeghi S., Macarulla T., Weiss K.H., Waldschmidt D.T., Goyal L., Borbath I., El-Khoueiry A. (2021). Infigratinib (BGJ398) in previously treated patients with advanced or metastatic cholangiocarcinoma with FGFR2 fusions or rearrangements: Mature results from a multicentre, open-label, single-arm, phase 2 study. Lancet Gastroenterol. Hepatol..

[42] Qi C., Shen L., Andre T., Chung H.C., Cannon T.L., Garralda E., Italiano A., Rieke D.T., Liu T., Burcoveanu D.I. (2025). Efficacy and safety of larotrectinib in patients with TRK fusion gastrointestinal cancer. Eur. J. Cancer.

[43] Doebele R.C., Drilon A., Paz-Ares L., Siena S., Shaw A.T., Farago A.F., Blakely C.M., Seto T., Cho B.C., Tosi D. (2020). Entrectinib in patients with advanced or metastatic *NTRK* fusion-positive solid tumours: Integrated analysis of three phase 1–2 trials. Lancet Oncol..

[44] Subbiah V., Kreitman R.J., Wainberg Z.A., Gazzah A., Lassen U., Stein A., Wen P.Y., Dietrich S., de Jonge M.J.A., Blay J.Y. (2023). Dabrafenib plus trametinib in BRAFV600E-mutated rare cancers: The phase 2 ROAR trial. Nat. Med..

[45] Subbiah V., Velcheti V., Tuch B.B., Ebata K., Busaidy N.L., Cabanillas M.E., Wirth L.J., Stock S., Smith S., Lauriault V. (2018). Selective RET kinase inhibition for patients with RET-altered cancers. Ann. Oncol..

[46] Subbiah V.A.-O., Cassier P.A., Siena S.A.-O., Garralda E., Paz-Ares L., Garrido P.A.-O., Nadal E.A.-O., Vuky J., Lopes G., Kalemkerian G.P. (2022). Pan-cancer efficacy of pralsetinib in patients with RET fusion-positive solid tumors from the phase 1/2 ARROW trial. Nat. Med..

[47] Yarlagadda B., Kamatham V., Ritter A., Shahjehan F., Kasi P.M. (2019). Trastuzumab and pertuzumab in circulating tumor DNA ERBB2-amplified HER2-positive refractory cholangiocarcinoma. NPJ Precis. Oncol..

[48] Ten Haaft B.H., Pedregal M., Prato J., Klumpen H.J., Moreno V., Lamarca A. (2024). Revolutionizing anti-HER2 therapies for extrahepatic cholangiocarcinoma and gallbladder cancer: Current advancements and future perspectives. Eur. J. Cancer.

[49] Harding J.J., Fan J., Oh D.-Y., Choi H.J., Kim J.W., Chang H.-M., Bao L., Sun H.-C., Macarulla T., Xie F. (2023). Zanidatamab for *HER2*-amplified, unresectable, locally advanced or metastatic biliary tract cancer (HERIZON-BTC-01): A multicentre, single-arm, phase 2b study. Lancet Oncol..

[50] Primrose J.N., Fox R.P., Palmer D.H., Malik H.Z., Prasad R., Mirza D., Anthony A., Corrie P., Falk S., Finch-Jones M. (2019). Capecitabine compared with observation in resected biliary tract cancer (BILCAP): A randomised, controlled, multicentre, phase 3 study. Lancet Oncol..

[51] Kelley R.K., Ueno M., Yoo C., Finn R.S., Furuse J., Ren Z., Yau T., Klumpen H.J., Chan S.L., Ozaka M. (2023). Pembrolizumab in combination with gemcitabine and cisplatin compared with gemcitabine and cisplatin alone for patients with advanced biliary tract cancer (KEYNOTE-966): A randomised, double-blind, placebo-controlled, phase 3 trial. Lancet.

[52] Lamarca A., Palmer D.H., Wasan H.S., Ross P.J., Ma Y.T., Arora A., Falk S., Gillmore R., Wadsley J., Patel K. (2021). Second-line FOLFOX chemotherapy versus active symptom control for advanced biliary tract cancer (ABC-06): A phase 3, open-label, randomised, controlled trial. Lancet Oncol..

[53] Marabelle A., Le D.T., Ascierto P.A., Di Giacomo A.M., De Jesus-Acosta A., Delord J.P., Geva R., Gottfried M., Penel N., Hansen A.R. (2020). Efficacy of Pembrolizumab in Patients With Noncolorectal High Microsatellite Instability/Mismatch Repair-Deficient Cancer: Results From the Phase II KEYNOTE-158 Study. J. Clin. Oncol..

[54] de Scordilli M., Bortolot M., Torresan S., Noto C., Rota S., Di Nardo P., Fumagalli A., Guardascione M., Ongaro E., Foltran L. (2025). Precision oncology in biliary tract cancer: The emerging role of liquid biopsy. ESMO Open.

[55] Moritz v W., Markus K., Eduard R. On the development of a flexible borescope fringe projection system. Proceedings of the SPIE.

[56] Wei-Hung S., Tzu-Chien H., Cho-Yo K. Three-dimensional shape measurements using endoscopes. Proceedings of the SPIE.

[57] Suksuratin P., Rodpai R., Luvira V., Intapan P.M., Maleewong W., Chuchuen O. (2022). Rapid label-free detection of cholangiocarcinoma from human serum using Raman spectroscopy. PLoS ONE.

[58] Wong C.Y., Seshadri P., Parks G.T. (2021). Automatic Borescope Damage Assessments for Gas Turbine Blades via Deep Learning. AIAA Scitech 2021 Forum.

[59] Troncoso D.A., Robles-Linares J.A., Russo M., Elbanna M.A., Wild S., Dong X., Mohammad A., Kell J., Norton A.D., Axinte D. (2023). A Continuum Robot for Remote Applications: From Industrial to Medical Surgery With Slender Continuum Robots. IEEE Robot. Autom. Mag..

[60] Zhang L., Pleskow D.K., Turzhitsky V., Yee E.U., Berzin T.M., Sawhney M., Shinagare S., Vitkin E., Zakharov Y., Khan U. (2017). Light scattering spectroscopy identifies the malignant potential of pancreatic cysts during endoscopy. Nat. Biomed. Eng..

[61] Du C., Chai N., Linghu E., Li H., Feng X., Wang X., Tang P. (2022). Diagnostic value of SpyGlass for pancreatic cystic lesions: Comparison of EUS-guided fine-needle aspiration and EUS-guided fine-needle aspiration combined with SpyGlass. Surg. Endosc..

[62] Wen Z., Dong Z., Deng Q., Pang C., Kaminski C.F., Xu X., Yan H., Wang L., Liu S., Tang J. (2023). Single multimode fibre for in vivo light-field-encoded endoscopic imaging. Nat. Photonics.

[63] Turtaev S., Leite I.T., Altwegg-Boussac T., Pakan J.M.P., Rochefort N.L., Cizmar T. (2018). High-fidelity multimode fibre-based endoscopy for deep brain in vivo imaging. Light. Sci. Appl..

[64] Stiburek M., Ondrackova P., Tuckova T., Turtaev S., Siler M., Pikalek T., Jakl P., Gomes A., Krejci J., Kolbabkova P. (2023). 110 mum thin endo-microscope for deep-brain in vivo observations of neuronal connectivity, activity and blood flow dynamics. Nat. Commun..

[65] George S.D.G., James J., Maria P.A., Travis S., Calum W., Catherine R.M.F., Philip H.J., Massimiliano di P., Rebecca C.F., Timothy D.W. (2019). Quantitative phase and polarization imaging through an optical fiber applied to detection of early esophageal tumorigenesis. J. Biomed. Opt..

[66] Jane C., George S.D.G. (2024). Ultra-miniature dual-wavelength spatial frequency domain imaging for micro-endoscopy. J. Biomed. Opt..

[67] Sweer J.A., Chen M.T., Salimian K.J., Battafarano R.J., Durr N.J. (2019). Wide-field optical property mapping and structured light imaging of the esophagus with spatial frequency domain imaging. J. Biophotonics.

[68] Angelo J.P., van de Giessen M., Gioux S. (2017). Real-time endoscopic optical properties imaging. Biomed. Opt. Express.

[69] Awe A.M., Rendell V.R., Lubner M.G., Winslow E.R. (2020). Texture Analysis: An Emerging Clinical Tool for Pancreatic Lesions. Pancreas.

[70] Shipp D.W., Sinjab F., Notingher I. (2017). Raman spectroscopy: Techniques and applications in the life sciences. Adv. Opt. Photon..

[71] Kong K., Rowlands C.J., Varma S., Perkins W., Leach I.H., Koloydenko A.A., Williams H.C., Notingher I. (2013). Diagnosis of tumors during tissue-conserving surgery with integrated autofluorescence and Raman scattering microscopy. Proc. Natl. Acad. Sci. USA.

[72] Boitor R., de Wolf C., Weesie F., Shipp D.W., Varma S., Veitch D., Wernham A., Koloydenko A., Puppels G., Nijsten T. (2021). Clinical integration of fast Raman spectroscopy for Mohs micrographic surgery of basal cell carcinoma. Biomed. Opt. Express.

[73] Boitor R.A., Varma S., Sharma A., Odedra S., Elsheikh S., Eldib K., Patel A., Koloydenko A., Gran S., De Winne K. (2024). Diagnostic accuracy of autofluorescence-Raman microspectroscopy for surgical margin assessment during Mohs micrographic surgery of basal cell carcinoma. Br. J. Dermatol..

[74] Corden C., Boitor R., Dusanjh P.K., Harwood A., Mukherjee A., Gomez D., Notingher I. (2023). Autofluorescence-Raman Spectroscopy for Ex Vivo Mapping Colorectal Liver Metastases and Liver Tissue. J. Surg. Res..

[75] Barkur S., Boitor R.A., Mihai R., Gopal N.S.R., Leeney S., Koloydenko A.A., Khout H., Rakha E., Notingher I. (2024). Intraoperative spectroscopic evaluation of sentinel lymph nodes in breast cancer surgery. Breast Cancer Res. Treat..

[76] Boitor R., Varma S., Sharma A., Elsheikh S., Kulkarni K., Eldib K., Jerrom R., Odedra S., Patel A., Koloydenko A. (2024). Ex vivo assessment of basal cell carcinoma surgical margins in Mohs surgery by autofluorescence-Raman spectroscopy: A pilot study. JEADV Clin. Pract..

[77] Mangiavillano B., Pagano N., Baron T.H., Luigiano C. (2015). Outcome of stenting in biliary and pancreatic benign and malignant diseases: A comprehensive review. World J. Gastroenterol..

[78] Song G., Zhao H.Q., Liu Q., Fan Z. (2022). A review on biodegradable biliary stents: Materials and future trends. Bioact. Mater..

[79] Lam R., Muniraj T. (2021). Fully covered metal biliary stents: A review of the literature. World J. Gastroenterol..

[80] Yang K., Sun W., Cui L., Zou Y., Wen C., Zeng R. (2024). Advances in functional coatings on biliary stents. Regen. Biomater..

[81] Suk K.T., Kim J.W., Kim H.S., Baik S.K., Oh S.J., Lee S.J., Kim H.G., Lee D.H., Won Y.H., Lee D.K. (2007). Human application of a metallic stent covered with a paclitaxel-incorporated membrane for malignant biliary obstruction: Multicenter pilot study. Gastrointest. Endosc..

[82] Chung M.J., Kim H., Kim K.S., Park S., Chung J.B., Park S.W. (2012). Safety evaluation of self-expanding metallic biliary stents eluting gemcitabine in a porcine model. J. Gastroenterol. Hepatol..

[83] Kim D.H., Jeong Y.I., Chung C.W., Kim C.H., Kwak T.W., Lee H.M., Kang D.H. (2013). Preclinical evaluation of sorafenib-eluting stent for suppression of human cholangiocarcinoma cells. Int. J. Nanomed..

[84] Kwak T.W., Lee H.L., Song Y.H., Kim C., Kim J., Seo S.J., Jeong Y.I., Kang D.H. (2017). Vorinostat-eluting poly(DL-lactide-co-glycolide) nanofiber-coated stent for inhibition of cholangiocarcinoma cells. Int. J. Nanomed..

[85] Wang H.W., Li X.J., Li S.J., Lu J.R., He D.F. (2021). Biliary stent combined with iodine-125 seed strand implantation in malignant obstructive jaundice. World J. Clin. Cases.

[86] Yang F., Ren Z., Chai Q., Cui G., Jiang L., Chen H., Feng Z., Chen X., Ji J., Zhou L. (2016). A novel biliary stent coated with silver nanoparticles prolongs the unobstructed period and survival via anti-bacterial activity. Sci. Rep..

[87] Yamabe A., Irisawa A., Kunogi Y., Kashima K., Nagashima K., Minaguchi T., Yamamiya A., Izawa N., Takimoto Y., Hoshi K. (2021). Development of biliary stent applying the antibacterial activity of silver: A literature review. Biomed. Mater. Eng..

[88] Park J.S., Yim K.H., Jeong S., Lee D.H., Kim D.G. (2019). A Novel High-Visibility Radiopaque Tantalum Marker for Biliary Self-Expandable Metal Stents. Gut Liver.

[89] Kobayashi M. (2019). Development of a biliary multi-hole self-expandable metallic stent for bile tract diseases: A case report. World J. Clin. Cases.

[90] Chen V.K., Eloubeidi M.A. (2005). Endoscopic ultrasound-guided fine-needle aspiration of intramural and extraintestinal mass lesions: Diagnostic accuracy, complication assessment, and impact on management. Endoscopy.

[91] Venkatachalapathy S.V., Aithal G.P. (2020). Endoscopic Ultrasound Sampling: From Cells to Tissue. Arch. Med. Health Sci..

[92] Hanna K., Krzoska E., Shaaban A.M., Muirhead D., Abu-Eid R., Speirs V. (2022). Raman spectroscopy: Current applications in breast cancer diagnosis, challenges and future prospects. Br. J. Cancer.

